# Role-Specific Brain Activations in Leaders and Followers During Joint Action

**DOI:** 10.3389/fnhum.2018.00401

**Published:** 2018-10-08

**Authors:** Léa A. S. Chauvigné, Steven Brown

**Affiliations:** Department of Psychology, Neuroscience & Behaviour, McMaster University, Hamilton, ON, Canada

**Keywords:** joint action, expertise, leading, following, dance, fMRI

## Abstract

Much of social interaction in human life requires that individuals perform different roles during joint actions, the most basic distinction being that between a leader and a follower. A number of neuroimaging studies have examined the brain networks for leading and following, but none have examined what effect prior expertise at these roles has on brain activations during joint motor tasks. Couple dancers (e.g., dancers of Tango, Salsa, and swing) are an ideal population in which examine such effects, since leaders and followers of partnered dances have similar overall levels of motor expertise at dancing, but can differ strikingly in their role-specific skill sets. To explore role-specific expertise effects on brain activations for the first time, we recruited nine skilled leaders and nine skilled followers of couple dances for a functional magnetic resonance imaging study. We employed a two-person scanning arrangement that allowed a more naturalistic interaction between two individuals. The dancers interacted physically with an experimenter standing next to the bore of the magnet so as to permit bimanual partnered movements. Together, they alternated between leading and following the joint movements. The results demonstrated that the brain activations during the acts of leading and following were enhanced by prior expertise at being a leader or follower, and that activity in task-specific brain areas tended to be positively correlated with the level of expertise at the corresponding role. These findings provide preliminary evidence that training at one role of a joint motor task can selectively enhance role-related brain activations.

## Introduction

Much joint action between two people involves the contrastive roles of leader and follower (Hasson and Frith, 2016). For example, when two people move a sofa, the front person is often the one who navigates the joint movement as well as the one who determines the speed at which the two people move, while the back person responds to these movement-cues and attempts to coordinate his/her actions with the front person. However, the experimental literature that examines joint action in the lab does not give consideration to individual differences, for example the fact that people may be predisposed toward being a leader or follower based on their personality traits or life experiences (Fairhurst et al., 2014). In typical studies of joint action, people are randomly assigned to being a leader or follower (or both) of a joint task without assessing individual differences in task expertise that may exist between them. This applies to studies of both experts (Goebl and Palmer, 2009; Sänger et al., 2012) and non-experts (Sacheli et al., 2013; Fairhurst et al., 2014; Konvalinka et al., 2014; Vesper and Richardson, 2014). This may be problematic since many studies demonstrate that expertise has an effect on behavioral performance and brain activations across many domains (Chang, 2014; Debarnot et al., 2014; Neumann et al., 2016).

An interesting solution to this problem is to examine couple dancers, such as Tango dancers, since such people engage in extensive training to develop expertise at one specific role in the dance, thereby making the assessment of leading/following experience on joint action quite feasible. Leaders and followers of a couple dance have similar overall levels of motor expertise at dancing, but they can differ strikingly in their role-specific skill sets, such that dancers of one role are often unable to dance the opposite role. This applies not merely to the movement patterns themselves, but to the *coordination* skills required for leading (e.g., force conveyance) and following (e.g., responsiveness to force cues). While previous neuroimaging studies have looked separately at the topics of leading/following and expertise, the current study–which is a follow-up analysis to a previously published study from our lab (Chauvigné et al., 2018)–represents a first attempt at examining *role-specific expertise* at leading and following, doing so using trained leaders and followers of couple dances. The principal aim of the study is to identify role-specific brain activations, namely leading-related activations in trained leaders compared to non-leaders, and following-related activations in trained followers compared to non-followers.

Previous studies of leading and following have tended to emphasize the networks for leading, more so than those for following. For example, studies of interactive imitation have compared the initiation and imitation of visual actions within the same group of participants, and have highlighted an initiation network involved in self-monitoring, willed action, and decision making (Chaminade and Decety, 2002; Decety et al., 2002; Nagy et al., 2010; Guionnet et al., 2012). Studies of auditory-entrainment tasks, such as finger tapping, have studied expert leaders or individuals who spontaneously emerge as leaders with in the context of the study, and have similarly identified a network involved in decision making, movement initiation, and self-processing (Sänger et al., 2012; Fairhurst et al., 2014; Konvalinka et al., 2014). These studies have provided either no results or inconsistent findings regarding following or expert followers. In a previous publication from our lab (Chauvigné et al., 2018), we characterized the networks for leading and following during a joint-action task with physical interaction, using the same dancer participants as those employed in the present study. In accordance with the previous literature, we found that leading showed a motor- and self-oriented profile, engaging areas associated with motor planning, spatial navigation, sequencing, action monitoring, and error correction. In contrast, following showed a far more sensory- and externally oriented profile, revealing areas involved in somatosensation, proprioception, motion tracking, social cognition, and outcome monitoring. However, while that study compared the act of leading with the act of following, it did not assess the influence of prior expertise at being a leader or follower on the brain activations. That was the major objective of the current follow-up analysis, namely to examine role-specific expertise.

It is well-established that expertise can influence both the structure and function of the brain. There is now a vast literature devoted to various forms of motor, perceptual, and cognitive expertise (reviewed in Bilalic, 2017). A general finding of such studies is that brain activations and gray matter volume are enhanced in experts, as compared to non-experts, in areas that process the skills that underlie a person’s domain of expertise (Chang, 2014; Debarnot et al., 2014; Neumann et al., 2016). For example, with regard to perceptual tasks, trained musicians and other auditory experts show enhanced effects in auditory cortex (Pantev et al., 2001, 2015; Margulis et al., 2009; Meyer et al., 2012), while visual experts show effects in visual cortex (Gauthier et al., 1999, 2000; Szwed et al., 2014). In the motor domain, effects are found in cortical and subcortical motor and premotor areas involved in motor execution, control, planning, and representation (Chang, 2014; Yang, 2015). Motor experts, such as athletes, dancers, and musicians, additionally demonstrate changes in perceptual and cognitive areas associated with their trained skills (Chang, 2014). For example, sensorimotor coupling is enhanced in musicians and athletes (Chang, 2014; Brown et al., 2015). In addition, activations in the action-observation network [including premotor cortex (PMC), superior parietal lobule (SPL), and inferior parietal lobule (IPL)] are enhanced when dancers view specific dance patterns that they are expert in (Calvo-Merino et al., 2005), or when athletes view sports actions that they are expert in (Balser et al., 2014), as compared to when the same people view dances or sports movements that they are not trained in. Calvo-Merino et al. (2006) suggested that this effect was due to motor training, rather than the associated perceptual training. Expertise, in addition to producing enhancements in processing, has also been linked to decreases in the overall number of activated foci in neuroimaging studies, especially in attentional and cognitive-control networks, suggesting an enhancement in automaticity of processing for the trained skill (Patel et al., 2013; Debarnot et al., 2014). The “two stage expertise hypothesis” (Guida et al., 2013; Debarnot et al., 2014) suggests that short-term training leads to enhancements of brain activations for the trained skill, while long-term training and skill mastering lead instead to decreases or reorganizations in brain activations.

While previous neuroimaging studies have looked at leading/following and expertise in isolation, no study thus far has combined the two issues, which is the principal objective of the present study. As mentioned above, couple dancers are an ideal cohort for exploring role-specific expertise in leading and following, since they spend many years developing expertise at typically just one of the two roles of the dance. As a result, expert leaders are usually unskilled followers, and vice versa, while both groups have comparable levels of overall motor expertise at the dance. More specifically, leader expertise during couple dancing requires the generation of a motor plan for both the self and the partner, and the efficient conveyance of signals to the partner, while follower expertise requires the tracking of information coming from the leader and its interpretation to construct either an identical or complementary movement pattern in real time.

In order to assess the effect of role expertise on brain activations during an ecologically valid joint-action task, we carried out an exploratory follow-up analysis to our previous publication that looked at leading and following (Chauvigné et al., 2018) in order to examine the effects of role-specific expertise on brain activations. In the previous study, skilled leaders and followers of couple dances performed both a leading and following task in a magnetic resonance imaging (MRI) scanner in interaction with an experimenter standing next to the bore of the magnet. The participant and experimenter were in physical contact at their hands, and alternated between being the leader and follower of joint improvised bimanual movements. The principal aim of the study was to compare brain activity during the acts of leading and following. The current study follows up on those results using the same dataset in order to examine the effects of individual differences on the brain activations, in particular an individual’s expertise at a given role of the dance. The aim was to look for role-specific brain activations, in other words leading-related activations in trained leaders compared to non-leaders (i.e., followers), and following-related activations in trained followers compared to non-followers (i.e., leaders). Based on the literature cited above demonstrating that experts show enhancements in task-specific brain areas compared to non-experts when performing the same tasks, we predicted that leaders, as compared with non-leaders, would show an enhancement of leading-related activations when leading (only), and likewise that followers, as compared with non-followers, would show an enhancement of following-related activations when following (only). Given that we were not able to effectively rule out the influence of gender on dance role in our design, the results need to be viewed as exploratory.

## Materials and Methods

### Participants

Eighteen participants (nine of each gender) took part in this study after giving their written informed consent in accordance with the Hamilton Integrated Research Ethics Board, who approved the study (St. Joseph’s Healthcare, R. P. #12-3777). They received monetary compensation for their participation. None of them had a past history of neurological or psychiatric disease. An inclusion criterion for the study was that participants have at least 2 years of experience at one or more kinds of couple dances involving leading and following (e.g., Argentine Tango, Salsa, Swing, and Ballroom). Male participants (40.7 ± 14.9 years old) had a mean dance experience of 8.7 ± 7.2 years, principally as leaders, although one male had significant experience as a follower as well. Female participants (40.2 ± 12.3 years old) had a mean dance experience of 5.6 ± 2.9 years, principally as followers, although two females had significant experience as leaders as well.

**FIGURE 1:**
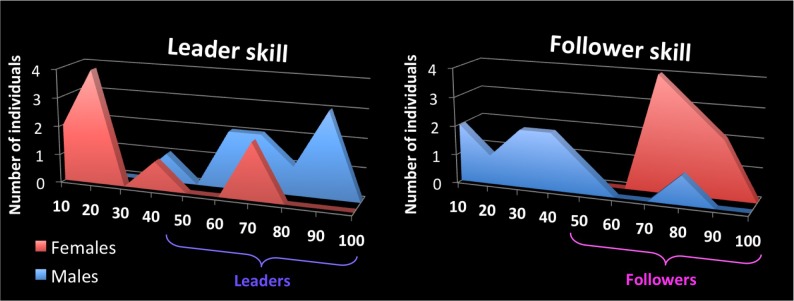
Self-report scales for skills as a leader and follower of couple dances. The *x*-axis shows the self-rating scale for leader skill (left panel) and follower skill (right panel) for couple dancing, where 100 is the highest rating. The *y*-axis of each graph shows the number of participants, from the pool of 18, who rated themselves at the various levels of skill for each role. Female participants are color-coded red and males are color-coded blue, both here and in **Figures 4, 5**. Participants designated as “leaders” in this study were comprised of all the males plus the two females with strong leading ability, while those designated as “followers” were comprised of all the females plus the male with strong following ability. Leaders are color coded as purple here and in **Figures 2–5**, whereas followers are color coded as pink here and in **Figures 2–5** (not to be confused with the color coding of gender).

On the day of the experiment, participants reported their ability to lead or follow a couple dance using a scale from 0 to 100, where 0 corresponds to no expertise at leading or following, and 100 corresponds to a very high level of expertise at leading or following. Each person did separate ratings for leading and following skill, with results shown in **Figure 1**. We explained to participants that these scales emphasized the ability to transmit/receive information while dancing with a partner, rather than the ability to perform complex or stylistic movements. Males reported a mean leading ability of 69.8 ± 17.7 (one male was at 35 and the rest ranged from 60 to 90). Likewise, females reported a mean following ability of 77.2 ± 8.3 (ranging from 70 to 90). With regards to the complementary skill, males reported a mean following ability of 33.7 ± 21.6; the male with significant following experience reported his following ability at 78, while all the others males rated it at between 8 and 50. Females reported a mean leading ability of 28.9 ± 25.2; both females with significant leading experience reported their leading ability at 70, while all other females rated themselves at between 5 and 40. Correlations between leading ability, following ability, years of experience at dancing, and age showed that leading ability, but not following ability, correlated with the number of years of experience (**Table 1**). Anecdotal evidence suggests that leading skill requires a greater amount of time and effort to achieve than does following skill, which may explain the exclusive correlation of leading skill with years of experience. Since leading and following ability were not anti-correlated in the analysis, participants designated as “leaders” in this study were comprised of all the participants who were primarily trained as leaders for at least 2 years (i.e., all the of males) plus the two participants who, although primarily trained as followers, had significant leading experience and a strong leading ability (two females). Those designated as “followers” were comprised of all the participants who were primarily trained as followers for at least 2 years (i.e., all of the females) plus one participant who, although primarily trained as leader, had significant following experience and a strong following ability (one male). Thus, three participants belonged in both groups. This division was used in only the first set of analyses (see below).

**Table 1:** Correlation between age, years of couple-dance experience, and self-reported leading and following skill.

	Age	Leading	Following	Years
Age	1	0.004 (*p* = 0.493)	0.101 (*p* = 0345)	0.436 (*p* = 0.035)
Leading		1	-0.343 (*p* = 0.082)	0.544 (*p* = 0.010)
Following			1	0.198 (*p* = 0.216)
Years				1


*The p-value is one-tailed*.

### Procedure

While the participant was lying supine in the MRI scanner, an experimenter (LASC) stood next to the bore of the scanner in order to have physical contact with the participant’s two hands. The participant’s forearms were fastened to the side of their body such that only their wrists, hands and fingers were able to move. Participants’ hands (palms up) were always below the experimenter’s hands (palms down), so that the participants’ hands could not be passively moved. The experimenter had significant experience both as a follower and a leader of couple dances. Together, the participant and experimenter performed highly controlled joint hand movements in all three planes of motion, alternating between leading and following the joint movement during different task-epochs of the scan. The movement patterns were improvised, rather than pre-learned, in order to maintain an ongoing requirement for motor planning during leading and a comparably heightened sense of responsiveness during following. No external cuing of tempo or rhythm was done with a metronome or with music. Participants performed all conditions with their eyes closed, and were instructed about which task to perform by means of pre-recorded verbal cues delivered through MRI-compatible headphones. Each condition was performed in a random order six times in blocks of 28 s.

Complete methods and details concerning fMRI acquisition and image analysis, including participant training, are described in Chauvigné et al. (2018). Briefly, the functional MRI imaging parameters were 2000 ms TR, 35 ms TE, 90° flip angle, 39 axial slices, 4 mm slice thickness, 0 mm gap, 3.75 × 3.75 mm in-plane resolution, 64 × 64 matrix, and 240 mm field of view. An automatic shimming procedure was performed before each scan to minimize inhomogeneities in the static magnetic field. For each of the three functional scans, 216 volumes–corresponding to 12 epochs of 28 s task + 8 s rest–were collected over 7’12”, resulting in a total of 648 volumes. Two magnetic field maps (5 ms then 8 ms TE) with the same imaging parameters as the fMRI were acquired in order to unwarp the data. Unwarping was performed with the relaxation method of “anatabacus”, a plugin in BrainVoyager, in order to correct for non-rigid deformations. In addition, the head-motion parameters were included as nuisance regressors in the analysis. Functional and structural images were processed using BrainVoyager QX 2.8. Coordinate tables were computed using NeuroElf.

### Analysis

We first performed qualitative analyses on three groups to assess if there were any differences between being a leader and being a follower. Specifically, we carried out three random-effects analyses for the bidirectional contrast “Leading versus Following” (1) for the whole group of 18 participants, (2) for the 11 leaders only, and (3) for the 10 followers only. These were performed at a two-tailed statistical threshold of *p* < 0.005 uncorrected with a cluster-level correction of *k* = 28 voxels determined with Alphasim (family-wise error *p* < 0.05) in NeuroElf. The conjunction of [Leading > Rest] ∩ [Following > Rest] was also performed on these three groups in order to serve as a reference for the general network of brain areas activated by the movement tasks, irrespective of role. It was performed at a two-tailed statistical threshold of *p* < 0.005 uncorrected with a cluster-level correction of *k* = 49 voxels determined with Alphasim.

Since qualitative differences were found (see Results section), we tested further for the effect of role by performing whole-brain regression analyses on the full group of participants (*n* = 18). We chose to perform statistical regression analyses instead of a direct statistical comparison between leaders and followers for two reasons. First, we consider role expertise to be a continuous trait, rather than a dichotomous one. Dancers can belong to both groups if they are trained at both leading and following. Thus a binary distinction would have led to a “male versus female” contrast, rather than a “leader versus follower” contrast. Second, the number of participants in each group was small (*n* = 10 and 11 for leaders and followers, respectively), whereas the regression involved the full group of 18 participants. Because of the small number of participants in the analysis and because of the small number of female leaders and male followers in the cohort, we consider this an exploratory study. Future studies will need to examine larger numbers of participants who have both leading and following skills, although such dual training tends to be limited to professional teachers of a dance.

For the whole-brain regression analyses, the self-reported values of leading and following skill were used as covariates in two separate analyses to regress the betas values of the contrast “Leading versus Following”. These regressions were also performed at a two-tailed statistical threshold of *p* < 0.005 uncorrected with a cluster-level correction of *k* = 25 voxels, determined with Alphasim. However, this threshold led to null results, and so we reported the activation at a less stringent threshold of *p* < 0.025 uncorrected with a cluster-level correction of k = 46 voxels, determined with Alphasim. We note that these results should be interpreted with caution and need to be replicated in future analyses. In order to examine the influence of gender, the mean beta value of each activated cluster was extracted for each participant and regressed against his/her corresponding leading or following skill.

## Results

**FIGURE 2:**
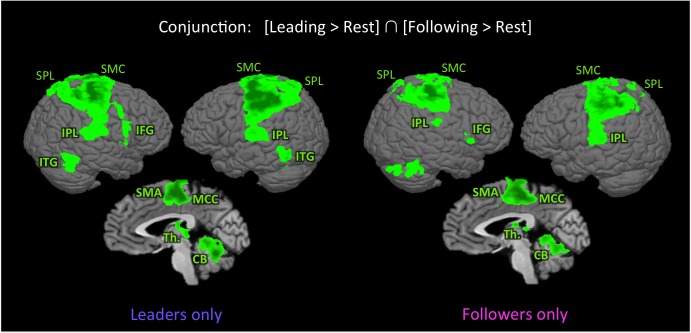
Shared network for leading and following. The figure shows the results of the conjunction [Leading > Rest] ∩ [Following > Rest] in leaders only (left panel) and followers only (right panel), *p* < 0.005 uncorrected (*k* = 49 voxels). With the exception of the inferior temporal gyrus, the activated network is similar in both followers and leaders. CB, cerebellum; IFG, inferior frontal gyrus; IPL, inferior parietal lobule; ITG, inferior temporal gyrus; MCC, middle cingulate cortex; SMA, supplementary motor area; SMC, sensorimotor cortex; SPL, superior parietal lobule; and Th., thalamus.

In order to identify the basic sensorimotor network involved in performing our joint bimanual tasks, we carried out the conjunction of [Leading > Rest] ∩ [Following > Rest], with results shown in **Figure 2** and Talairach coordinates reported in **Table 2**. This shared network between leading and following consisted of a widespread sensorimotor cortical (primary motor and somatosensory cortex) and subcortical (thalamus and cerebellum) network, as well as the supplementary motor area (SMA), midcingulate cortex (MCC), SPL, inferior frontal gyrus (IFG), IPL (including the secondary somatosensory cortex [SII] and extending to the insula), and inferior temporal gyrus (ITG), extending to the middle temporal gyrus (MTG). Except for the ITG, which was present in leaders only, this network was found in both leaders and followers.

**Table 2:** The shared network for leading and following.

			Leaders only (*n* = 11)					Followers only (*n* = 10)				
				
Area	BA	Hemisphere	*x*	*y*	*z*	*k*	Max	*x*	*y*	*z*	*k*	Max
SMC	1,3,4,5,6,40	RH	33	-40	58	1356	17.01	30	-37	61	1149	16.54
SMC	2,3,4,5,6,40	LH	-39	-43	52	1419	17.67	-39	-37	55	1135	18.19
IFG	6,13,44	RH	54	2	19	174	5.86	54	8	7	108	8.05
SMA/MCC	6, 24, 31	RH/LH	3	-13	52	610	14.68	0	-22	49	683	12.14
IPL/SII	13,40,41	RH	48	-28	25	294	7.26	45	-34	31	108	6.82
IPL/SII	13, 22,40	LH	-51	-28	19	240	9.52	-48	-34	19	314	9.36
SPL	7	RH	24	-61	58	197	7.06	24	-61	55	166	6.52
SPL	7	LH	-24	-61	58	453	10.52	-27	-55	58	268	9.76
ITG	37	RH	51	-58	-8	117	6.45					
ITG	19, 37	LH	-48	-58	4	93	6.30					
Thalamus		RH	15	-25	10	105	7.03	0	-16	16	49	6.73
Thalamus		LH	-15	-19	10	139	7.41					
Cerebellum	Vermis	RH/LH	-3	-61	-17	308	10.74	3	-58	-14	163	7.72
Cerebellum	Culmen/Declive	RH	15	-49	-17	164	9.53	12	-46	-17	104	6.19
Cerebellum	Culmen/Declive	LH	-21	-49	-20	140	10.20	-15	-52	-17	144	8.87
Cerebellum	Tuber/Declive	RH	42	-58	-23	94	7.03	45	-58	-20	156	7.86


*Talairach coordinates for the conjunction of Leading and Following compared to rest (*p* > 0.005 uncorrected, *k* = 49 voxels). IFG, inferior frontal gyrus; IPL, inferior parietal lobule; ITG, inferior temporal gyrus; SII, secondary somatosensory cortex; SMA, supplementary motor area; SMC, sensorimotor cortex; and SPL, superior parietal lobule.*

**FIGURE 3:**
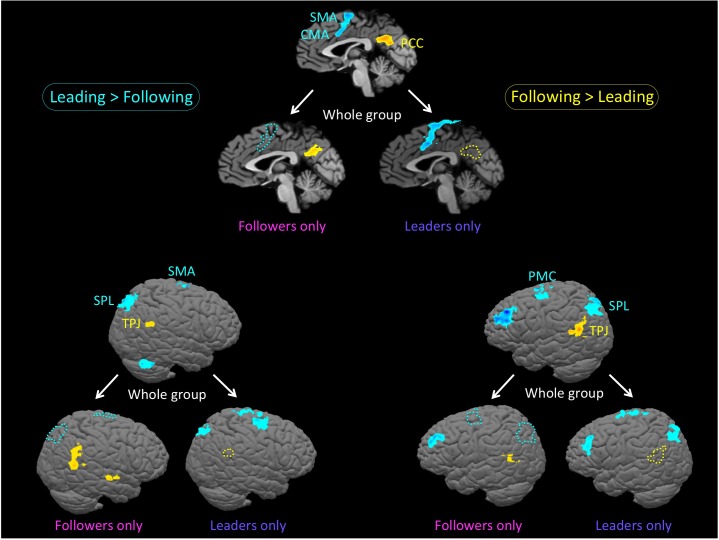
Role-specific brain activations. The figure shows an analysis of the bidirectional “Leading versus Following” contrast in three groupings: the whole group of 18 participants; only the leaders (a subset of 11 participants); and only the followers (a subset of 10 participants). Contrasts are performed at *p* < 0.005 uncorrected (*k* = 28 voxels). The top panel is the midsagittal view, the lower left panel is the left hemisphere, and the lower right panel is the right hemisphere. Each panel is set up as a triad, with the whole group at the top and the restricted analyses of leaders-only and followers-only below that. Cyan clusters and outlines reflect the contrast of “Leading > Following”, whereas yellow clusters and outlines reflect the reverse contrast of “Following > Leading”. In order to facilitate the visualization of role-specific activations, we use colored outlines to represent whole-group activations that are missing in either the leaders-only or the followers-only analyses. More specifically, cyan outlines are regions of whole-group activation that are present in the leaders-only analysis, but not the followers-only analysis, while yellow outlines are regions of whole-group activation that present in the followers-only analysis, but not the leaders-only analysis. The leaders-only analysis shows the same network for leading as the whole group, but no brain areas for following. The followers-only analysis shows the same network for following as the whole group, but only the cerebellum and dorsolateral prefrontal cortex for leading. Only role-specific activations are labeled in this figure. Leading network: CMA: cingulate motor area; PMC, premotor cortex; SMA, supplementary motor area; and SPL, superior parietal lobule. Following network: PCC, posterior cingulate cortex and TPJ, temporo-parietal junction.

We next wanted to explore our question of interest, namely whether there was evidence for role-specific activations, in other words activations found only in skilled individuals while performing the role they are trained in. This would reveal whether leaders and followers engage different brain resources during leading and following. As shown in **Figure 3** and **Table 3**, we first qualitatively compared three types of analyses of the “Leading > Following” contrast (cyan clusters) and “Following > Leading” contrast (yellow clusters): the whole group of 18 participants; only the leaders (a subset of 11 participants); and only the followers (a subset of 10 participants). Overall, the leaders-only analysis showed basically the same network for leading as the whole group, but no brain areas for following. Likewise, the followers-only analysis showed basically the same network for following as the whole group, but only the dorsolateral prefrontal cortex (DLPFC) for leading (Note that only role-specific activations are labeled in the **Figure 3**).

**Table 3:** Leading versus following in the whole group, the leader-only group, and the follower-only group.

			Whole-group (*n* = 18)					Leaders only (*n* = 11)					Followers only (*n* = 10)				
					
Area	BA	Hemisphere	*x*	*y*	*z*	*k*	Max	*x*	*y*	*z*	*k*	Max	*x*	*y*	*z*	*k*	Max
**Activations: Leading > Following**																	
pre-SMA	6	RH/LH						3	5	58	29	5.24					
SMA	4,6	RH/LH	-4	-4	59	81	5.97	-3	-13	64	109	6.28					
CMA	24	RH/LH	-6	8	37	47	4.84	0	2	40	85	8.41					
PMC	6	RH						21	-4	55	145	8.01					
PMC	6	LH	-26	-13	54	51	4.36	-30	-16	64	32	5.71					
DLPFC	8,9	LH	-40	29	38	98	7.35	-48	32	31	46	5.84	-39	44	34	76	7.08
DLPFC	9	LH						-36	23	25	30	6.91					
SPL	7	RH	6	-73	42	70	6.39	6	-73	49	47	7.56					
SPL	7	LH	-16	-73	36	84	5.44	-18	-79	43	48	6.34					
Cerebellum	Tuber	RH	47	-65	-17	37	5.00										
**Deactivations: Following > Leading**																	
PCC	7,31	RH	3	-54	23	129	-5.36						3	-61	31	92	-11.12
TPJ	39,40	RH	45	-59	25	28	-5.33						48	-61	31	89	-8.86
TPJ	39,40	LH	-53	-63	23	105	-5.70										
STS	19,39	RH											48	-61	16	92	-10.18
STS	37,39	LH											-51	-52	4	66	-5.94
aSTG	13,22	RH											57	-16	-2	72	-9.70
Temporal pole	38	LH											-48	5	-23	30	-8.09
PHC	30,36	RH	17	-33	0	39	-4.72						42	-40	1	28	-6.13
PHC	28	LH	-21	-16	-6	103	-5.76						-30	-22	-11	67	-7.52
Thalamus		RH											21	-25	4	59	-6.72


*Talairach coordinates for the contrast “Leading versus Following” in the whole group, leaders only, and followers only (*p* > 0.005 uncorrected, *k* = 28 voxels). aSTG, anterior superior temporal gyrus; CMA, cingulate motor area; DLPFC, dorsolateral prefrontal cortex; PCC, posterior cingulate cortex; PHC, parahippocampal cortex; PMC, premotor cortex; SMA, supplementary motor area; SPL, superior parietal lobule; STS, superior temporal sulcus; and TPJ, temporo-parietal junction.*

Regarding the leading task, role-specific activations that were found exclusively in skilled leaders (cyan activations in **Figure 3** in both the leaders-only and whole-group brains that correspond with the cyan outlines in the followers-only brain) were observed in the SMA and cingulate motor area (CMA; top panel), SPL (right and left hemispheres in the lower panels), and PMC(left hemisphere). In addition, while leading, leaders showed a more extended premotor activation than the whole-group, especially in the right hemisphere (**Table 3**).

Regarding the following task, role-specific activations that were found exclusively in skilled followers (yellow activations in **Figure 3** in both the followers-only and whole-group brains that correspond with the yellow outlines in the leaders-only brain) were observed in the posterior cingulate cortex (PCC; top panel), temporo-parietal junction (TPJ; right and left hemispheres in the lower panels), and parahippocampal cortex (PHC, not shown). In addition, while following, followers showed activity in the posterior superior temporal sulcus (pSTS) that was not present in the whole group (**Table 3**). To summarize, the networks associated with leading and following seemed to be more strongly engaged by experts at the corresponding role than non-experts at that role.

**FIGURE 4:**
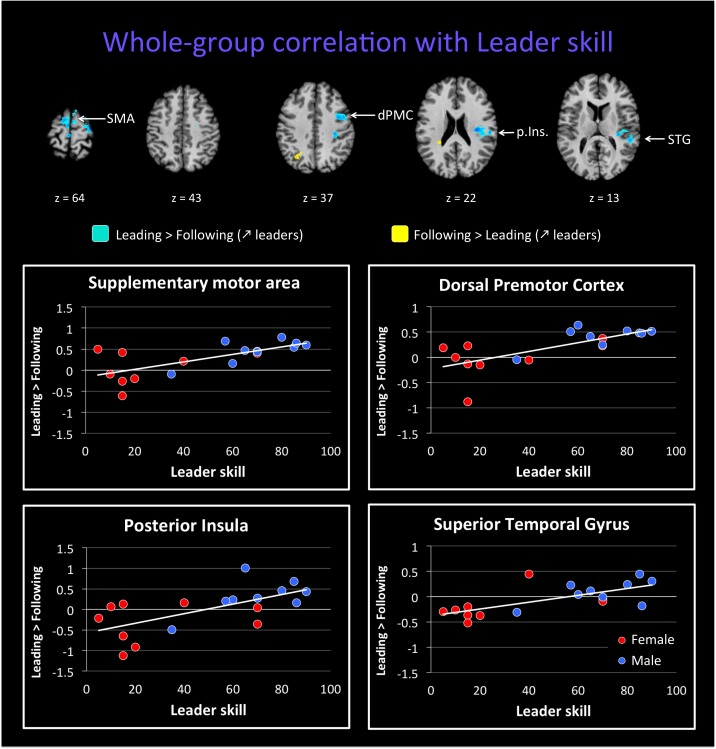
Regression of brain activation with leader skill in the whole group of participants. The top panel of this figure shows brain activity that correlates with the contrasts “Leading > Following” (cyan activations) and “Following > Leading” (yellow activations). Contrasts are performed at *p* < 0.02 uncorrected (*k* = 25 voxels). Brain areas for “Leading > Following” that correlate with leader skill include the dorsal premotor cortex (dPMC), insula (Ins.), superior temporal gyrus (STG), and supplementary motor area (SMA). Almost no areas for the contrast “Following > Leading” correlate with leader skill (see **Table 4**). The lower plots show mean beta values extracted from the SMA, dPMC, posterior insula and STG against leader skill, where female participants are shown with red dots and male participants with blue dots. Activity for leading increased with increasing leader skill, and this seems to be independent of gender.

**FIGURE 5:**
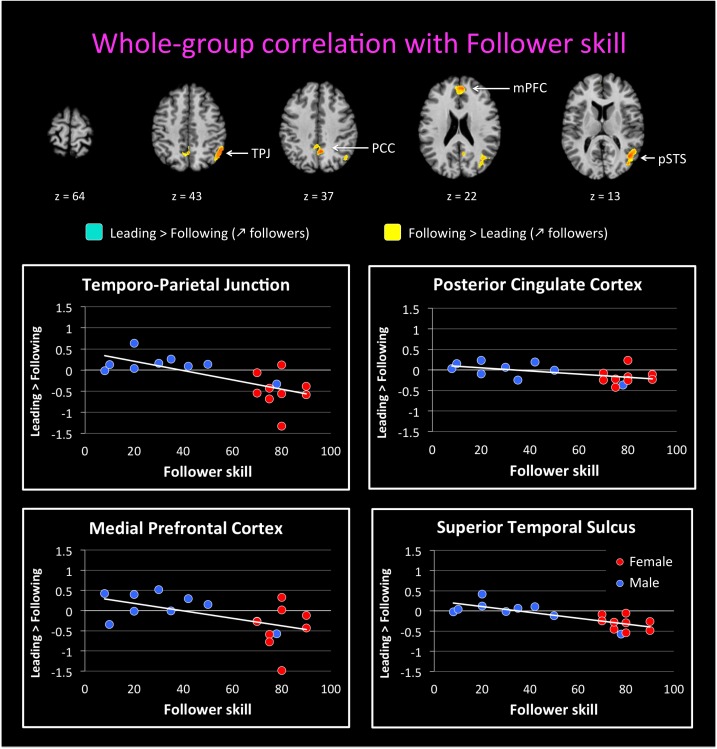
Regression of brain activation with the follower skill in the whole group of participants. The top panel of this figure shows brain activity that correlates with the contrasts “Leading > Following” (cyan activations) and “Following > Leading” (yellow activations). Contrasts are performed at *p* < 0.02 uncorrected (*k* = 25 voxels). Brain areas for “Following > Leading” that correlate with follower skill include the medial prefrontal cortex (mPFC), posterior parietal cortex (PCC), posterior superior temporal sulcus (pSTS), and temporo-parietal junction (TPJ). No areas appeared for the contrast “Leading > Following” correlate with follower skill (see **Table 5**). The lower plots show mean beta values extracted from the TPJ, PCC, mPFC, and pSTS against follower skill, where female participants are shown with red dots and male participants with blue dots. Activity for following increased with increasing follower skill, and this seems to be independent of gender.

We followed up on these qualitative analyses with whole-brain regressions in which the self-reported expertise at being a leader or follower (see **Figure 1** above) was used as the covariate for the contrast of leading versus following. Activations for these analyses were only found at a more lenient threshold, but are still reported since they are consistent with both our hypotheses and the qualitative analyses reported above. However, the results should be interpreted with caution. **Figure 4** shows the regressions with leader skill, and **Figure 5** shows the regressions with follower skill. The regions where activations during the leading task correlated with leader skill included the SMA, pre-SMA, dorsal PMC (dPMC), superior temporal gyrus (STG), and insula (**Figure 4** top panel, **Table 4**). The regions where activations during the following task correlated with follower skill include the PCC, TPJ, pSTS, and mPFC (**Figure 5** top panel, **Table 5**). For each cluster, the coefficient of determination (*R*^2^) of the regression of the mean beta value against leader and follower skill is shown in **Tables 4, 5**, respectively.

**Table 4:** Leading versus following correlated with skill as a leader.

	Area	BA	Hemisphere	*x*	*y*	*z*	*k*	Max	*R*^2^
**Activation**									
	pre-SMA	6	RH	6	8	58	55	0.73	0.37
	SMA	6	RH	12	-7	55	184	0.81	0.46
	dPMC	6	RH	36	-1	37	46	0.66	0.49
	Insula	13	RH	33	-25	28	101	0.71	0.50
	STG	41,22	RH	54	-28	10	62	0.66	0.48
**Deactivation**									
	Cingulate	13,13	LH	-21	-34	28	87	-0.74	0.37
	Lingual	19	LH	-33	-58	-2	46	-0.72	0.30


*Talairach coordinates for the contrast “Leading versus Following” in the whole group correlated with the covariate “leader skill” (*p* > 0.025 uncorrected, *k* = 46 voxels). *R*^2^ is the coefficient of determination of the regression of the cluster’s mean beta value against leader skill. dPMC, dorsal premotor cortex; SMA, supplementary motor area; and STG, superior temporal cortex.*

**Table 5:** Leading versus following correlated with skill as a follower.

	Area	BA	Hemisphere	*x*	*y*	*z*	*k*	Max	*R*^2^
**Activation**									
**Deactivation**	PCC	7,31	RH/LH	6	-55	37	78	-0.75	0.30
	mPFC	9,10	RH/LH	6	47	22	64	-0.73	0.27
	pSTS	19,39	RH	42	-61	13	90	-0.75	0.61
	TPJ	39,40	RH	48	-52	43	72	-0.72	0.48


*Talairach coordinates for the contrast “Leading versus Following” in the whole group correlated with the covariate “follower skill” (*p* > 0.025 uncorrected, *k* = 46 voxels). *R*^2^ is the coefficient of determination of the regression of the cluster’s mean beta value against follower skill. mPFC, medial prefrontal cortex; PCC, posterior cingulate cortex; pSTS, superior temporal sulcus; and TPJ, temporo-parietal junction.*

Examples of how the mean beta value in these regions covaries with leader and follower skill are shown in the bottom panels of **Figures 4, 5**, respectively. The results provide some evidence that activity in these regions might depend on the level of expertise. However, they in no way rule out a gender effect, either alone or in interaction with expertise, and so the results have to be seen as preliminary. In the dPMC and STG (**Figure 4**, bottom panels), activity for the contrast of “Leading > Following” increased with leader skill, but a male with low leader skill had a low activity, whereas females with high leader skill had a high activity. Other areas that correlated with leader skill had the same trend (not shown). Similarly, in the mPFC and TPJ (**Figure 5**, bottom panels), activity for the contrast “Leading > Following” decreased with follower skill (that is, “Following > Leading” activity increased with follower skill), but a male with high follower skill had a low activity, similar to females with high follower skill. Other areas that correlated with follower skill had the same trend (not shown). Future studies will be needed to fully exclude the influence of gender on the expertise effects observed here. Hence, the current study must be seen as a pilot study that gives a first glimpse at role-specific expertise effects without being able to effectively factor out the influence of gender.

## Discussion

This current exploratory study examined for the first time the effect of expertise at the coordinative skills involved in leading and following on brain activations during a joint-action task in a realistic setting. Its results provide support for the existence of role-specific brain activations during joint actions. In particular, we observed that leading-related activations were enhanced in leaders compared to followers when both groups performed the leading task, and that following-related activations were enhanced in followers compared to leaders when both groups performed the following task. Additionally, we showed that leading-related brain regions in the whole group of participants tended to correlate with expertise at being a leader, whereas following-related brain regions tended to correlate with expertise at being a follower. Another way of conceptualizing these results is that the skilled leaders hardly engaged any areas during following that were not already engaged during leading; likewise, the skilled followers hardly engaged any areas during leading that were not already engaged during following. This might explain the null results found in some previous studies when comparing following with leading (Fairhurst et al., 2014). These results suggest that expertise at one role of a joint-action task can enhance brain activations for the trained role compared to the untrained role. Hence, not only do the results support the existing literature on expertise effects for motor tasks, but they extend it for the first time to the contrastive roles of leader and follower in joint actions.

The major finding of the initial qualitative analysis (**Figure 2**) was that the brain networks that we observed for leading and following in the whole group seemed to be mainly supported by prior experience at being a leader or follower. In particular, skilled followers strongly engaged the mentalizing and social networks (PCC, TPJ, and STS) while following, which is consistent with a view of following as a process of adapting to one’s partner or as inferring knowledge from one’s partner (Saxe, 2006; Lieberman, 2007; Konvalinka et al., 2010; Mar, 2011; Sacheli et al., 2013; Vesper and Richardson, 2014). In contrast, skilled leaders strongly engaged networks for motor control and planning (SMA, CMA, PMC, and cerebellum) and for spatial navigation and exploration (SPL) while leading, which is consistent with the requirements of the leading role (Glover, 2004; Brown et al., 2006; Sänger et al., 2012; Fairhurst et al., 2014; Konvalinka et al., 2014). Interestingly, both skilled leaders and skilled followers activated the DLPFC during leading, which implies that self-initiation and action selection (Frith, 2000; Bengtsson et al., 2007; Guionnet et al., 2012) are probably the most important characteristics of leading, regardless of expertise.

By performing whole-brain regressions with leading or following skill, we treated being a leader or follower as a continuous trait, rather than a dichotomous one. Although we did not find any activity using our *a priori* threshold, the activations observed at a more lenient threshold were consistent with both our hypotheses and the qualitative results, and are thus reported as exploratory findings. We observed that distinct brain areas tended to correlate with the level of self-reported expertise at being a leader or a follower, respectively. The areas that correlated with follower skill were principally components of the following network, such as the mPFC, PCC, TPJ, and pSTS. Thus, the more that someone is trained at following, the more that s/he will recruit brain regions of the mentalizing and social networks, which might indicate more attention to, or more efficient processing of, social stimuli (i.e., cues coming from the leader) and the mental states of others (i.e., their intentions and action plans). Another characteristic of followers is their ability to track their partner’s movements or other signaling cues so as to produce either imitative or complementary movements. Along these lines, the pSTS has been specifically implicated in the multisensory perception of biological motion (Grossman et al., 2005; Kavounoudias et al., 2008), indicating that a trained follower might be specialized in analyzing information coming from the partner’s movement, not least haptic information emanating from body contact (Chauvigné et al., 2017).

In contrast to this profile for following, the areas that tended to correlate with leader skill were mainly part of the leading network, including premotor areas (pre-SMA, SMA, and PMC). Other areas that tended to correlate with leader skill were the insula and STG. This network is quite similar to the one shown to be activated by motor experts in the meta-analysis of Yang (2015). In addition, all of the areas associated with leader skill in the present study have been previously shown to be involved in improvisation (Beaty, 2015). Since leading requires the ability to improvise movements, we can assume that the better a person is at leading, the better s/he can improvise a motor plan for both the self and the partner, and thus the more s/he recruits premotor areas and the STG. However, it has also been shown that improvisational expertise (in musicians, for example) is related to a deactivation in the DLPFC, TPJ, IFG, and insula (Berkowitz and Ansari, 2010; Pinho et al., 2015), which has been interpreted as indicating an automation of cognitive processing and a greater focus on internal processes during improvisation (Beaty, 2015). The absence of deactivations in these regions in our study can potentially be explained by the fact that our use of a joint task may have precluded the adoption of an internal focus by the participants when leading. Indeed, a study of joint improvisation also found an activation increase in the DLPFC, pre-SMA, and STG (Donnay et al., 2014), which is quite similar to a situation of improvising with a dance partner when leading.

Overall, the study integrates two issues in the cognitive neuroscience of motor performance, first the contrast between leading and following, and second the influence of individual differences in motor expertise on brain activations. As mentioned in the Introduction, many experimental studies of joint action randomly assign people to being a leader or follower of a joint task (Goebl and Palmer, 2009; Sänger et al., 2012; Sacheli et al., 2013; Vesper and Richardson, 2014; Fairhurst et al., 2014). However, in Western dance culture, people are generally assigned these roles based on their gender, with men tending to be assigned the role of leader in couple dances. Thus, in contrast to a study of piano duetting (Goebl and Palmer, 2009), for example, people come to a dance study like ours with years of experience at just one role of the joint task. This provides us with the unique ability to examine individual differences in joint action based not on random factors but on role-specific training. Previous studies of expertise processing have demonstrated enhanced brain activations in experts compared to non-experts (Gauthier et al., 1999, 2000; Pantev et al., 2001, 2015; Margulis et al., 2009; Meyer et al., 2012; Debarnot et al., 2014; Chang, 2014; Szwed et al., 2014; Neumann et al., 2016; Bilalic, 2017). However, this has often has been investigated using non-motor tasks, even in motor experts like professional ballet dancers (Calvo-Merino et al., 2005). We have instead probed this using a motor task, with the added benefit of doing this using a joint-action task. The integration of these two issues is that we were able to examine the contrast between leading and following–as per studies of joint action–but to incorporate the factor of prior motor experience, as per studies of expertise processing. The results revealed a clear overlap between these two issues, such that the brain activations during the acts of leading and following were enhanced by prior expertise at being a leader or follower, and that activity in task-specific brain areas tended to be positively correlated with the level of expertise at the corresponding role. In other words, we were able to demonstrate *role-specific enhancements* in brain activation.

### Limitations

Given that this study was a first attempt to examine the effect of role expertise on brain activations during joint action, we are aware that it has a number of significant limitations. First, we were limited in our ability to measure behavioral performance during task production in the scanner due to an absence of MRI-compatible technologies such as motion capture at our imaging center. Thus, we cannot determine if the differences between leaders and followers seen in the study are due to trait-related differences in activation or behavioral differences as well. The joint-action task performed in this study was quite simple and involved very small hand movements. Hence, it did not require any type of specialized skill, which would foster similar performance in the two groups. In addition, the experimenter was the sole interaction partner for all of the participants in the study and was thus a controlled factor in the interaction. However, the absence of a technology like motion capture means that we are unable to rule out behavioral differences between participants as a source of the results. Further research taking advantage of MRI-compatible technologies will be required to explore this issue.

Second, the qualitative analyses showed an interesting pattern that was confirmed by the whole-group regression at a more lenient threshold, but not at a standard threshold. Hence, the effects seem to be small. Although the observed activations at the less stringent threshold were consistent with our expectations based on previous studies, the results of this study should be taken with caution and need to be replicated, preferentially with a larger cohort and a wider spread of skill levels. In addition, the skill levels that were used to regress the brain data were self-report data. They might thus have been subject to self-report biases and inaccuracies. However, no objective measure of leadership and/or followership skills exists in the literature. Given the preliminary results of this study, it would be worthwhile to develop such measures in future. Such measures could be used to see if the results of the present study could be replicated based on people’s role expertise in some other motor skill outside of dancing, or even on people’s natural predispositions to be a leader or follower, as related to personality traits and life experiences, rather than the specialized skill of dance training.

Finally, and importantly, we are unable to rule out gender as a factor in determining the role-specific effects in our study, and hence the results need to be seen as quite preliminary. While the leader and follower groups were not exclusively of one gender, they did have a majority of one gender. Given the evidence for gender effects on a diversity of perceptual, cognitive, and motor tasks (Cosgrove et al., 2007; Hines, 2010; Gur et al., 2012; Ingalhalikar et al., 2014; Hyde, 2016), further studies will be required to assess a gender contribution to our results with trained couple dancers. Given the paucity of female leaders and male followers in the world of couple dancing, perhaps the only approach that will be able to address the limitations of the current study is a training study. A study that crosses gender with role during a several-month training program of leading or following for some joint-action task could permit a disentangling of the relative effects of gender and expertise. If female leaders and male followers showed the same role-specific effects as in the current study, this would argue against a gender interpretation in favor of expertise *per se*. Such a study could also reveal potential gender effects as well.

## Conclusion

This study is the first to look at the influence of prior individual training at being a leader or follower on the brain activations occurring during the acts of leading and following, thereby assessing the effect of role expertise during naturalistic joint action. Our major finding was that leaders and followers do not seem approach leading and following in the same way at the neural level, with leaders engaging more brain resources during leading, and followers during following, thus reflecting role-specific activations. Additionally, we showed that activity in leading-related brain regions tended to correlate with expertise at being a leader, and likewise that activity in following-related brain regions tended to correlate with expertise at being a follower. These findings highlight the fact that the acts of leading and following might be skill-specific, and thus that prior experience at these roles should be assessed when studying leading and following during joint action. However, given our inability to disentangle gender from dance role, the current results must be seen as preliminary. A training study that crosses gender with role will probably be required to truly distinguish dance role from gender.

## Author Contributions

LC ran the experiment and analyzed the data. LC and SB conceived the experiment, analyzed the results, and wrote the manuscript.

## Conflict of Interest Statement

The authors declare that the research was conducted in the absence of any commercial or financial relationships that could be construed as a potential conflict of interest.
